# Seronegative limbic encephalitis in association with Sjögren's syndrome: a rare case report

**DOI:** 10.11604/pamj.2022.41.52.28790

**Published:** 2022-01-19

**Authors:** Sabu George, Neena Baby, Percival Gilvaz, Harisuthan Thangheswaran, Mary Anne Poovathingal, Alex Baby

**Affiliations:** 1 Department of Neurology, Jubilee Mission Medical College Hospital and Research Institute, Thrissur, Kerala, India,; 2 Department of Neurology, Renai Medicity Multi Super Speciality Hospital, Kochi, Kerala, India

**Keywords:** Limbic, encephalitis, plasmapheresis, Sjögren syndrome, case report

## Abstract

Limbic encephalitis is often due to an autoimmune or paraneoplastic disease and is always a diagnostic challenge. We report a 31-year-old lady who presented with fever and proximal weakness to start with and afterwards developed refractory focal onset seizures and worsening cognition despite optimum treatment. Evaluation revealed hypokalemia with a normal anion gap metabolic acidosis. Magnetic resonance imaging (MRI) brain showed features of limbic encephalitis. Cerebrospinal fluid (CSF) showed lymphocytosis and CSF autoimmune, paraneoplastic and viral encephalitis panel were negative. However a blood ANA profile clinched the diagnosis when SS-A and Ro 52 were strongly positive. She was given steroids and subsequently plasma exchange. A labial gland biopsy confirmed the diagnosis of Sjögren syndrome. In cases of autoimmune limbic encephalitis with no identifiable cause, serological screening for rheumatological disorders is recommended. Sjögren syndrome is a rare aetiology for autoimmune limbic encephalitis. A detailed history and a step wise approach is always the key to the right diagnosis.

## Introduction

Limbic encephalitis can be due to an infective or non-infective etiology. The common non infective etiologies are autoimmune, paraneoplastic or metabolic causes. Limbic encephalitis (LE) affects predominantly the medial temporal lobes [1]. The usual presentation is with cognitive impairment, psychiatric changes, and seizures. Autoimmune limbic encephalitis has always been a diagnostic challenge. Sjögren syndrome (SS) is a rare aetiology for autoimmune limbic encephalitis and there have been only a very few case reports in literature so far [2,3]. Often a detailed history, and a step wise approach may give a clue to the clinical syndrome.

## Patient and observation

**Patient description:** a 31-year-old lady presented with history of low grade fever followed by generalized weakness which was predominantly proximal and symmetrical, of one-week duration. She had a past history of hypokalemic weakness two years ago in her postpartum period, which improved with symptomatic treatment. During the present episode she had fever, increased thirst and urinary frequency of 1 week duration, which was treated as urinary infection. On examination she was confused, with a Glasgow coma scale of E4V3M5, pupils were equal and reactive with normal fundi. She was moving all 4 limbs and there were no signs of meningeal irritation.

**Figure 1:**
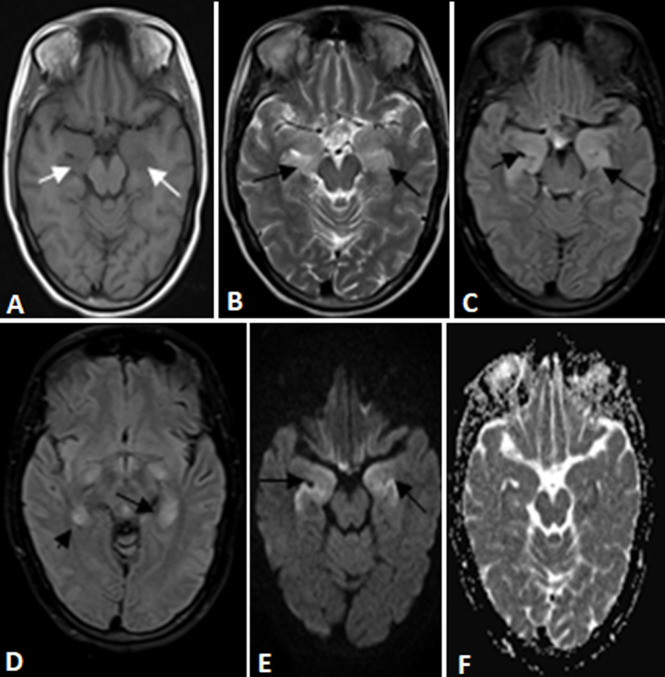
magnetic resonance imaging of brain showing hypointensity over bilateral medial temporal lobes on T1 weighted images (panel A marked with white arrows); hyperintensity over bilateral medial temporal region on T2 weighted (panel B); FLAIR sequences (panel C and D marked with black arrows) with no diffusion restriction; (panel E, diffusion weighted images and F, apparent diffusion coefficient images)

**Diagnostic assessment:** blood investigations showed normal anion gap metabolic acidosis, hypokalemia, normal sodium levels. Her serum potassium level was 2.7mEq/l and was started on intravenous correction along with oral supplementation. Hemogram, renal and thyroid function test, calcium, magnesium, viral markers and Anti thyroid peroxidase antibody were within normal limits. However, her erythrocyte sedimentation rate (ESR) was 43 mm/hr and on serial testing had mild thrombocytopenia. A computed tomogram (CT) brain was taken in view of her worsening cognition, and it was normal. Arterial blood gas showed hyperchloremic metabolic acidosis with normal anion gap (AG). Serum cortisol and aldosterone levels were normal. Overall picture was suggestive of distal renal tubular acidosis (RTA) considering acidosis, polyuria and hyperechoic kidneys on ultrasound abdomen and she was started appropriate treatment. A magnetic resonance image (MRI) of brain was done which showed T2 and FLAIR hyperintensities involving both medial temporal lobes without any diffusion restriction, and minimal contrast enhancement suggestive of limbic encephalitis (Figure 1). Subsequently she developed intermittent focal onset seizures involving mainly the face with twitching of eyelids, lips and right upper limb. Electroencephalogram showed occasional left temporal spikes. However, she was oriented and obeying in between the seizure episodes, with no focal deficits, thus ruling out a status epilepticus. Cerebrospinal fluid (CSF) study showed lymphocytic pleocytosis with 20 cells (P5, L95) with no hypoglycorrhachia and mildly elevated proteins (62mg/dl). Cerebrospinal fluid (CSF) polymerase chain reaction (PCR) for meningoencephalitis panel was negative for bacteria and viruses. Her blood and CSF culture were sterile. CSF and serum for autoimmune aetiologies including NMDA-R antibody, LGI1, CASPr2, GAD-65, GABABR, Anti Hu, Ri, Ma-2, were all negative.

**Figure 2:**
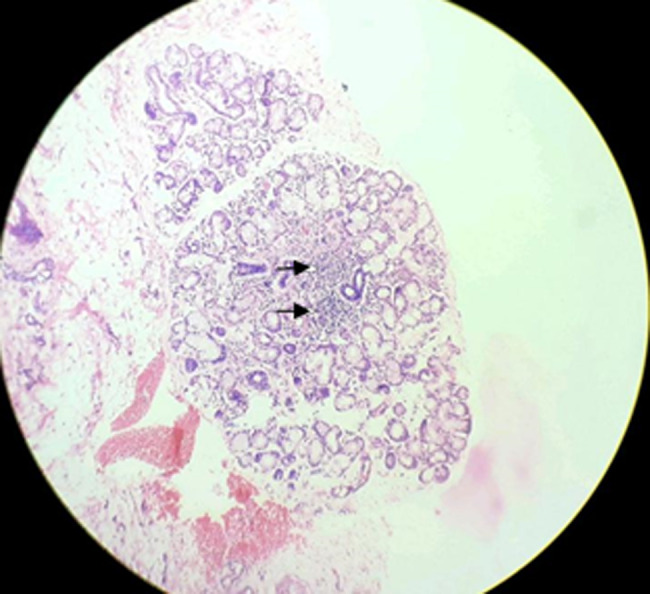
showing hematoxylin and eosin (H&E) staining of salivary gland lobule with 2 foci of more than 50 lymphocytes (black arrow) and occasional plasma cell

**Therapeutic intervention:** she was started on intravenous methyl prednisolone. But she continued to have recurrent focal seizures despite on four antiepileptic medications in adequate doses (oxcarbazepine, valproate, clobazam and perampanel). In view of her inadequate response to steroids, 5 cycles of plasma exchange were done. Her paraneoplastic work up including fluorodeoxyglucose (FDG)-positron emission tomography (PET) scan was non-contributory. On reassessing her, in view of polydypsia, polyuria, hypokalemia, normal anion gap metabolic acidosis (NAGMA) a rheumatological syndrome was considered. Antinuclear antibody (ANA) profile study offered the clue to diagnosis when SS-A and Ro-52 were strongly positive. Her vasculitic workup including ANCA antibodies were negative. She started showing signs of improvement after 3^rd^ cycle of plasma exchange. Seizure frequency reduced, and cognition improved with no focal deficits. As our patient did not have sicca symptoms of eyes, Schirmer test was not performed. A labial gland biopsy showed lymphoid cells infiltration satisfying Focus score (Figure 2). Presence of increased thirst, positive SS-A and Focus score of >1 confirmed the diagnosis of Sjögren syndrome satisfying 2016 American College of Rheumatology and the European League Against Rheumatism (ACR-EULAR) criteria. Anticonvulsants were gradually tapered. She was discharged on immunomodulation therapy, oral prednislone and mycophenolate mofetil (MMF) 500mg twice daily. Oral steroids were slowly tapered and stopped. She was continuing on MMF and was asymptomatic at 6-months follow up.

**Patient consent:** informed consent obtained for publication from patient on follow up visit.

## Discussion

Limbic encephalitis (LE) can be due to paraneoplastic or non paraneoplastic cause (autoimmune, infective, metabolic). However, there have been only very few reports of LE in Sjögren syndrome. Sjögren syndrome (SS) is characterised by lymphocytic infiltration and destruction of the exocrine glands. Nervous system manifestations occur in a quarter of patients and more commonly peripheral sensory neuropathy or mononeuritis multiplex [4]. Brain and spinal cord involvement is rare but brainstem syndrome, cerebellar syndrome and transverse myelitis have been reported.

A significant decrease in salivary, lacrimal secretion, inflammatory infiltrates in salivary gland by lip biopsy and the presence of anti-SS-A and anti-SS-B antibodies are included in ACR-EULAR diagnostic criteria for SS. Cognitive dysfunction and psychiatric symptoms were the outstanding features of encephalitis associated with primary SS in most patients [5]. An extensive search for an underlying malignancy and alternative autoimmune, infectious and metabolic aetiologies were done in our patient and subsequently, the diagnosis was established as autoimmune LE associated with SS.

Advances in neuroimmunology research have led to the identification of new clinical syndromes and biomarkers that have transformed the diagnostic approach to autoimmune encephalitis [6]. However, diagnostic criteria for autoimmune limbic encephalitis include: A subacute onset of memory deficits, psychiatric symptoms of less than 3-months, MRI FLAIR changes restricted to bilateral medial temporal lobes, CSF pleocytosis or epileptiform discharges from temporal lobe with no alternate diagnosis. Even in cases with minimal rheumatologic findings, antibody screening for rheumatologic disorders including Sjögren syndrome should be performed in all cases of suspected paraneoplastic syndromes. It can be a game changer. Although LE associated with Sjögren syndrome may have a severe and progressive course, it seems to have a dramatic response to immunotherapy [5,7,8]. It is henceforth, important not to overlook the diagnosis of SS and consider immunosuppressive treatment, especially if there are no other identifiable causes to account for the presentation. There is also an outside chance that our patient might be having autoimmune encephalitis, with as yet uncharacterized/unidentified anti-neuronal antibodies related to Sjögren syndrome. Nevertheless, contrast enhancement, high incidence of inflammatory CSF findings and a good response to immunotherapy with minimal or no residual deficits might suggest that LE related to Sjögren syndrome may constitute a distinct syndrome.

## Conclusion

Limbic encephalitis, more often is due to an autoimmune, paraneoplastic or infective aetiology and has been a challenge both in the diagnosis and management. However in cases of autoimmune limbic encephalitis with no identifiable cause, serological screening for rheumatologic disorders is warranted. Sjögren syndrome though a rare aetiology for autoimmune limbic encephalitis, should not be missed out. It can make the difference between treatable and hopeless.

## References

[1] Graus F, Titulaer MJ, Balu R, Benseler S, Bien CG, Cellucci T (2016). A clinical approach to diagnosis of autoimmune encephalitis. The Lancet Neurology.

[2] Çoban A, Özyurt S, Meriç K, Misirli H, Tüzün E, Türkoğlu R (2016). Limbic encephalitis associated with Sjögren's syndrome: report of three cases. Internal Medicine.

[3] Finelli PF, Inoa V (2013). Limbic encephalitis as the presenting feature of Sjögren syndrome. Neurology: Clinical Practice.

[4] Delalande S, De Seze J, Fauchais AL, Hachulla E, Stojkovic T, Ferriby D (2004). Neurologic manifestations in primary Sjögren syndrome: a study of 82 patients. Medicine.

[5] Moreira I, Teixeira F, Silva AM, Vasconcelos C, Farinha F, Santos E (2015). Frequent involvement of central nervous system in primary Sjögren syndrome. Rheumatology international.

[6] Abboud H, Probasco JC, Irani S, Ances B, Benavides DR, Bradshaw M (2021). Autoimmune encephalitis: proposed best practice recommendations for diagnosis and acute management. Journal of Neurology, Neurosurgery & Psychiatry.

[7] Budhram A, Leung A, Nicolle MW, Burneo JG (2019). Diagnosing autoimmune limbic encephalitis. CMAJ.

[8] Ismail II, Alnaser F, Al-Hashel JY (2021). Seronegative limbic encephalitis manifesting as subacute amnestic syndrome: a case report and review of the literature. Journal of Medical Case Reports.

